# An improved approach to infer protein-protein interaction based on a hierarchical vector space model

**DOI:** 10.1186/s12859-018-2152-z

**Published:** 2018-04-27

**Authors:** Jiongmin Zhang, Ke Jia, Jinmeng Jia, Ying Qian

**Affiliations:** 1 Department of Computer Science & Technology, East China Normal University, North Zhongshan Road, Shanghai, 200062 China; 2 School of life science, East China Normal University, Dongchuan Road, Shanghai, 200241 China

**Keywords:** Protein-protein interaction, Gene Ontology, Vector space model, Functional similarity

## Abstract

**Background:**

Comparing and classifying functions of gene products are important in today’s biomedical research. The semantic similarity derived from the Gene Ontology (GO) annotation has been regarded as one of the most widely used indicators for protein interaction. Among the various approaches proposed, those based on the vector space model are relatively simple, but their effectiveness is far from satisfying.

**Results:**

We propose a Hierarchical Vector Space Model (HVSM) for computing semantic similarity between different genes or their products, which enhances the basic vector space model by introducing the relation between GO terms. Besides the directly annotated terms, HVSM also takes their ancestors and descendants related by “is_a” and “part_of” relations into account. Moreover, HVSM introduces the concept of a Certainty Factor to calibrate the semantic similarity based on the number of terms annotated to genes. To assess the performance of our method, we applied HVSM to Homo sapiens and Saccharomyces cerevisiae protein-protein interaction datasets. Compared with TCSS, Resnik, and other classic similarity measures, HVSM achieved significant improvement for distinguishing positive from negative protein interactions. We also tested its correlation with sequence, EC, and Pfam similarity using online tool CESSM.

**Conclusions:**

HVSM showed an improvement of up to 4% compared to TCSS, 8% compared to IntelliGO, 12% compared to basic VSM, 6% compared to Resnik, 8% compared to Lin, 11% compared to Jiang, 8% compared to Schlicker, and 11% compared to SimGIC using AUC scores. CESSM test showed HVSM was comparable to SimGIC, and superior to all other similarity measures in CESSM as well as TCSS. Supplementary information and the software are available at https://github.com/kejia1215/HVSM.

## Background

The Gene Ontology (GO) [1] is a widely used vocabulary system in bioinformatics, which systematically describes the functional relations between different genes or their products. The GO consists of three independent ontologies: biological process (BP), cellular component (CC), and molecular function (MF). Each ontology is structured as a Directed Acyclic Graph (DAG), in which GO terms form the nodes, and the relations between the GO terms form the edges. In the DAG, GO terms are connected by different hierarchical relations (mostly is_a and part_of relations). The is_a relation describes the fact that a child term is a specialization of a parent term, while the part_of relation denotes the fact that a child term is a component of a parent term. The term at the lower level (e.g., leaf term) has more specific information than the term at the upper level (e.g., root term). Recently, GO has been widely used in protein function prediction, validation [2, 3] and classification of protein-protein interactions [4, 5], gene expression studies [6] and pathway analysis [7].

Gene products are usually annotated with a set of GO terms. The functional relations between gene products are quantified by using the shared GO terms of gene products [8–10] or explicitly using semantic similarity measures [11]. The semantic similarity measures have been widely used, which generate numerical values describing the likeness between two terms [12].

In this paper we presented a new method to calculate semantic similarity, the Hierarchical Vector Space Model (HVSM), which enhanced the basic vector space model (VSM) by explicitly introducing the relations between GO terms. When constructing the vector for a gene, in addition to the terms annotated to the gene, HVSM takes their ancestors and descendants into consideration as well. Besides, HVSM considers both “is_a” and “part_of” relations. The introduction of the Certainty Factor to calibrate the similarity value based on the number of annotated terms improves the effectiveness of HVSM further. The simplicity of the algorithm makes it very efficient. We tested HVSM on *Homo sapiens* and *Saccharomyces cerevisiae* protein-protein interaction datasets and compared the results with two other vector-based measures, IntelliGO [13] and basic VSM, and the six other popular measures, including TCSS [14], Resnik [15], Lin [16], Jiang [17], Schlicker [18], and SimGIC [19]. The results showed that HVSM outperformed the other eight measures in most cases. HVSM achieved an improvement of up to 4% compared to TCSS, 8% compared to IntelliGO, 12% compared to VSM, 6% compared to Resnik, 8% compared to Lin, 11% compared to Jiang, 8% compared to Schlicker, and 11% compared to SimGIC. The correlation coefficients with protein sequence, EC, and Pfam similarity also showed that HVSM was comparable to SimGIC, and outperformed all other similarity measures in the CESSM test.

### Related Work

Different approaches have been proposed to calculate the semantic similarity, such as the vector-based approach, the term-based approach, the set-based approach, and the graph-based approach. The vector-based approach transforms a gene product into a vector, and functional similarity is measured by the similarity of corresponding vectors. The term-based approach calculates semantic similarities from term similarities using various combination strategies. The set-based approach views the set of terms as bags of words. Two gene products are similar if there is a large overlap between the two corresponding sets of terms. The graph-based approach uses graph matching techniques to compute the similarity.

In vector-based approaches, the dimension of the vector is equal to the total number of terms in GO. Each dimension corresponds to a term in GO. Each vector component is either 1 or 0, denoting the presence or absence of a term in the set of annotations of a given gene product. The alternative way is to have each dimension represent a certain property of a term (e.g., IC value) [20]. The most common method of measuring similarity between vectors is the cosine similarity:

1 $$\begin{array}{@{}rcl@{}} S_{v}\left(G_{1},G_{2}\right)=\frac{v_{1}\cdot v_{2}}{\left|v_{1}\right|\left|v_{2}\right|} \end{array} $$

where *v*_*i*_ represents the vector of the gene product *G*_*i*_, *v*_1_·*v*_2_ corresponds to the dot product between the two vectors, and |*v*_*i*_| denotes the magnitude of vector *v*_*i*_.

Suppose *G*_1_ and *G*_2_ are two given genes or gene products annotated by two sets of GO terms {*t*_11_,*t*_12_,⋯,*t*_1*n*_} and {*t*_21_,*t*_22_,⋯,*t*_2*m*_}. IntelliGO [13], a vector-based method, represented each gene as a vector $g=\sum _{i}\alpha _{i}e_{i}$, where *α*_*i*_=*w*(*g*,*t*_*i*_)*IFA*(*t*_*i*_), *w*(*g*,*t*_*i*_) representing the weight assigned to the evidence code between *g* and *t*_*i*_, *IFA*(*t*_*i*_) being the inverse annotation frequency of the term *t*_*i*_, and *e*_*i*_ being the *i*-th basis vector corresponding to the annotation term *t*_*i*_. The dot product between two gene vectors was defined as:

2 $$\begin{array}{@{}rcl@{}} g_{1}*g_{2}=\sum_{ij}\alpha_{i}*\beta_{i}*e_{i}*e_{j} \end{array} $$

3 $$\begin{array}{@{}rcl@{}} e_{i}*e_{j}=\frac{2Depth(LCA)}{MinSPL\left(t_{1i},t_{2j}\right)+2Depth(LCA)} \end{array} $$

where *Depth*(*LCA*) was the depth of the lowest common ancestor (LCA) for *t*_1*i*_ and *t*_2*j*_, and *MinSPL*(*t*_1*i*_,*t*_2*j*_) was the length of the shortest path between *t*_1*i*_ and *t*_2*j*_, which passed through *LCA*. The similarity measure for the two genes vectors *g*_1_ and *g*_2_ was then defined using the cosine formula:

4 $$\begin{array}{@{}rcl@{}} {SIM}_{IntelliGO}\left(g_{1},g_{2}\right)=\frac{g_{1} \cdot g_{2}}{\sqrt{g_{1}*g_{1}}\sqrt{g_{2}*g_{2}}} \end{array} $$

The basic vector-based methods ignore the intrinsic relationship between different terms and treat different terms as independent components, which may lead to the inaccuracy of the semantic similarity.

Term-based approaches can be classified into two groups: path-based and IC-based.

Path-based approaches, also called edge-based approaches [2, 21–26], use the number of edges or the distance between two terms to quantify the semantic similarity. When more than one path exist between two terms, the shortest path or the average of all paths is usually used. Similar approaches were adapted to the biomedical field [27]. Path-based methods are based on two assumptions: (1) edges and nodes are uniformly distributed [28], and (2) edges at the same level in the ontology correspond to the same semantic distance between terms. However, both of the above assumptions are rarely true.

IC-based approaches [14–19, 29–32] use the Information Content (IC) to measure how specific and informative a term is. IC can be quantified by negative log likelihood, − log*p*(*c*), where *p*(*c*) is the occurrence probability of the term *c* in a specific corpus, such as the UniProt Knowledge base [12]. The TCSS [14] measure defined a different way to calculate IC, which depended upon the specificity of the term in the graph, shown as:

5 $$\begin{array}{@{}rcl@{}} ICT(t)=-ln\left(\frac{\left|{N(t)}\right|}{\left|{O}\right|}\right) \end{array} $$

where *t* was a term in the ontology *O*, |*N*(*t*)| was the number of children terms of *t*, and |*O*| was the total number of terms in *O*. The IC value of a term was dependent on its children, and its parents were not considered [15].

Many of the term-based methods are hybrid. They involve both ideas of the path-based and IC-based approaches, so the distinction between the two groups is not clear. Three combination approaches are commonly used in term-based approaches to obtain semantic similarities of gene pairs from term similarities: maximum (MAX), average (AVG) and best-match average (BMA) [18]. Let GO(A) and GO(B) denote the term sets annotated to two proteins A and B. The MAX and the AVG approach are given by the maximum and the average of the similarity between each term in GO(A) and each term in GO(B). The BMA is given by the average similarity between each term in GO(A) and its most similar term in GO(B), averaged with its reciprocal [33].

Set-based approaches use the Tversky ratio model of similarity [34] (a general model of distance) to calculate the similarity between gene products, which is defined as:

6 $$\begin{array}{@{}rcl@{}} \frac{f\left(G_{1}\cap G_{2}\right)}{f\left(G_{1}\cap G_{2}\right)+\alpha*f\left(G_{1}-G_{2}\right)+\beta*f(G_{2}-G_{1})} \end{array} $$

where *G*_1_ and *G*_2_ are sets of terms annotated to two different gene products from the same ontology and *f* is an additive function on sets. When *α*=*β*=1, we get the Jaccard distance between two sets:

7 $$\begin{array}{@{}rcl@{}} S_{Jaccard}=\frac{f\left(G_{1}\cap G_{2}\right)}{f\left(G_{1}\cup G_{2}\right)} \end{array} $$

When $\alpha =\beta =\frac {1}{2}$, we have the Dice distance between two sets:

8 $$\begin{array}{@{}rcl@{}} S_{Dice}=\frac{2*f\left(G_{1}\cap G_{2}\right)}{f\left(G_{1}\right)+f\left(G_{2}\right)} \end{array} $$

Set-based approaches assume that the terms are independent of each other. The similarity and dissimilarity of genes are modeled by two sets and their interactions. From Eqs. (7) and (8), we can conclude that the Jaccard and Dice distance return a similarity of 0 if two sets have no shared terms. However, these terms may have a certain relationship in the GO hierarchy.

Graph-based approaches make use of graph matching and graph similarity to calculate the similarity between gene products. A gene is modeled by the sets of nodes and edges associated with a sub-graph. The similarity is calculated by quantifying the difference between two sub-graphs.

Graph-based methods have three disadvantages: (1) a few measures only takes into account the shared terms in the sub-graphs, ignoring the edge type [35–38]; (2) graph matching have a weak correlation with similarity between terms [39]; (3) graph matching is an NP-complete problem [40].

Mazandu et al. [11] compared fourteen semantic similarity tools based on GO, classified in the context of IC models, term similarity approaches and functional similarity measures. The features and challenges of each approach were analyzed, including the use scope and limitations. Mazandu et al. also described two key reasons for the difficulty in comparison: the dataset issue, where different tools use different version of GO or annotation datasets, and the scaling issue, which results from tools making different assumption regarding normalization methods.

The effects of the shared information for the semantic similarity calculation were discussed in [41]. The shared information of a term pair is the common inheritance relations extracted from the structure of the GO graph. Experiments of three different methods calculating the term similarity, each with five shared information methods, were done on three ontologies across six benchmarks. Among the choice of shared information, term similarity algorithm, and ontology type, the choice of ontology type most strongly influenced the performance, and shared information type had the least influence [41].

More and more hybrid approaches were proposed in recent years, such as the algorithm described in [42], which utilized both the topological features of the GO graph and the information contents of the GO terms. Based on the topological structure of the GO graph, the measure [42] identified a number of GO terms as cluster centers according to a specific threshold, and then a membership was calculated for each cluster center and term pair. Semantic similarity scores were obtained by combining the relevant memberships and shared information contents. The threshold and the width of the Gaussian membership function were determined for different ontologies and datasets respectively to achieve the best AUC scores, while most of the other methods, including TCSS, used fixed value of parameters. Besides, the normalization method used in [42] depended on different ontologies. Therefore, the method showed relatively good performance.

The machine learning approaches are emerging to study semantic similarity, such as support vector machine (SVM) [43], random forest [44], and AdaBoost strategy [45]. Among the machine learning techniques, random forest and support vector machine (SVM) are found to achieve the best performance [43].

Methods involving natural language processing were reported. w2vGO [46] utilized the Word2vec model to compare definitions of two GO terms, which did not rely on the GO graph. The results showed that w2vGO was comparable to Resnik [15].

The semantic similarity measure was also extended to gene network analysis. GFD-Net [47] combined the concept of semantic similarity with the use of gene network topology to analyze the functional dissimilarity of gene networks based on GO. It was used in gene network validation to prove its effectiveness.

## Methods

We propose the HVSM algorithm, which is based on the Vector Space Model, to calculate the semantic similarity between genes. Similar to basic VSM approaches, HVSM maps each gene into a vector, and the semantic similarity between two genes is obtained by calculating the similarity between two corresponding vectors. The key improvement of HVSM over basic VSM lies in the refinement of the vector generation. When transforming the set of terms annotated to a gene to a vector, HVSM considers the relations between terms in the hierarchy structure of the GO graph. HVSM takes into account not only each directly annotated GO term, but also their ancestors and descendants, which are related by “is_a” and “part_of” relations. Thus, vectors in HVSM represent the attributes of genes more comprehensively and accurately, compared with basic VSM.

Figure 1 shows the main procedure of HVSM, which consists of four stages.

1. Initialize the vectors. Each vector component is binary valued, with 1 representing the presence of the GO term in the gene’s annotation and 0 representing its absence;

**Fig. 1:**
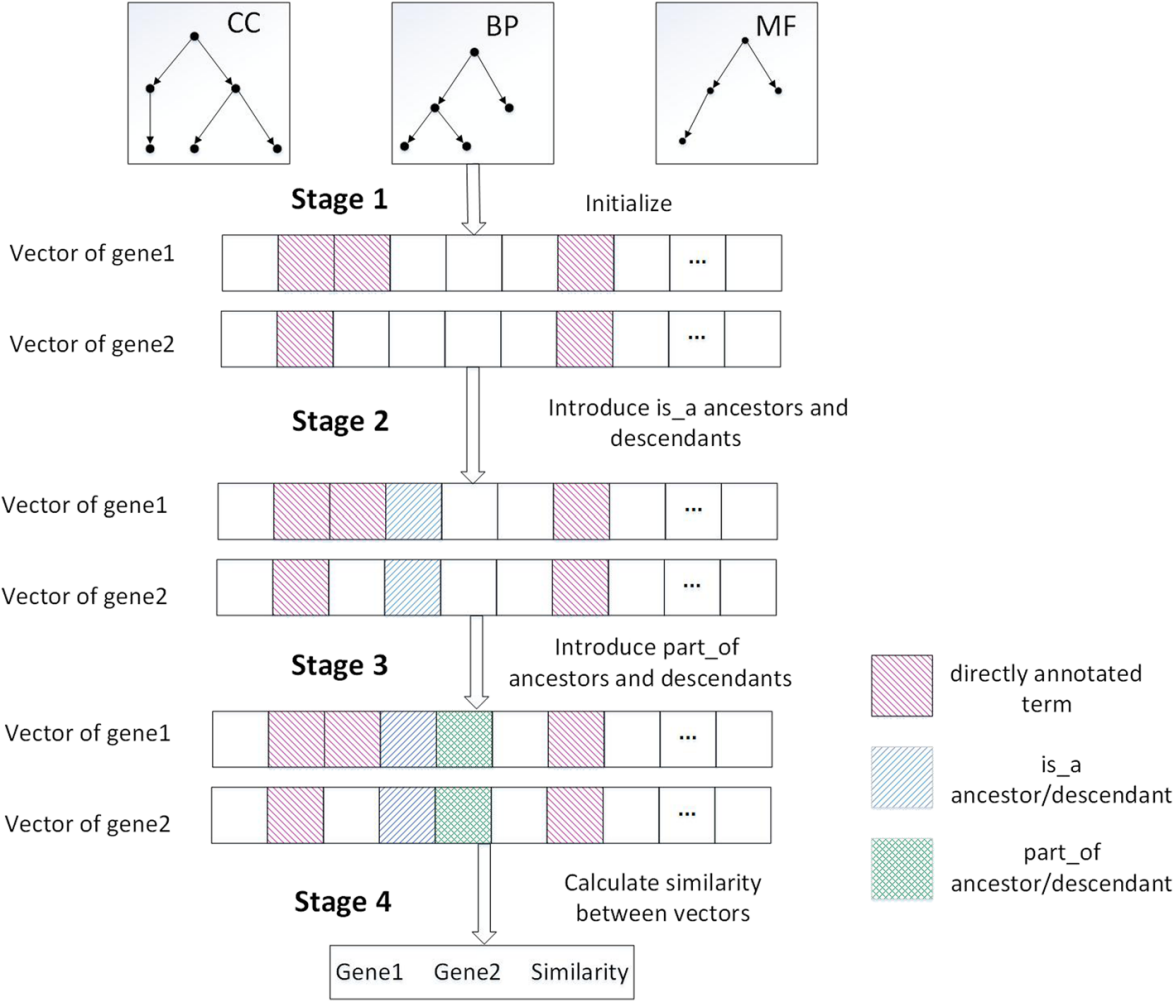
The main process of HVSM
2. Find out the parents and children of the directly annotated terms via “is_a” relations and then modify the vector accordingly;
3. Find out the parents and children of the directly annotated terms via “part_of” relations and then modify the vector accordingly;
4. Calculate similarity between vectors enhanced with the certainty factor.

In stage 1, each gene has a set of directly annotated terms and each element in the set denotes a functional aspect of the gene. The dimension of the vectors generated by vector-based methods, including the HVSM, equals the total number of terms in GO, with each dimension corresponding to a specific term in GO. Each component value of the vector represents the relative degree of the contribution of the corresponding terms. Thus, the vector generated for a gene represents the function distribution of the gene. Let *n* be the dimension of the vector. The vector *g* for a given gene *G* can be denoted as $g=\left (t_{1}^{G},t_{2}^{G},\cdots,t_{n}^{G}\right)$, where $t_{i}^{G}$ has value between 0 and 1, which reflects the relevance of term *i* to gene *G*.

The main steps of stage 2 of HVSM are described in detail as follows:

i. Deal with parents.
  For the directly annotated terms, their parents are considered individually. For each parent, if the value of the component corresponding to a parent is 0, we add the value *w*_*parent*_∗*w*_*is*_*a*_ to it, where *w*_*parent*_ and $w_{is\_a}$ are the semantic contribution factors for parent terms and “is_a” relation, respectively. If the value of the component corresponding to a parent is equal to 1, the value remains unchanged. If it is between 0 and 1, we add *w*_*incre*_∗*w*_*is*_*a*_ to it, where *w*_*incre*_ is the increment factor for shared nodes. The modified value of the component should not be larger than 1. Let $t_{i}^{G^{\prime }}$ be the value of *i*th component corresponding to a parent. The value after modification, $t_{i}^{G}$, is expressed as:
  9 $$ {}t_{i}^{G}= \left\{ \begin{array}{ll} w_{parent}*w_{is\_a}& {t_{i}^{G^{\prime}}=0;}\\ min\left(1,{t_{i}^{G^{\prime}}}+{w_{incre}*w_{is\_a}}\right)& {t_{i}^{G^{\prime}}\neq0;} \end{array}\right.  $$
ii. Deal with grandparents.
  The grandparent terms are considered with a similar strategy to that used in step i for the parent terms. We introduce $w_{r\_g}$, which is the ratio of contribution factor for grandparents. The value of *i*th component corresponding to a grandparent after modification, $t_{i}^{G}$, is expressed as:
  10 $$ {}t_{i}^{G}= \left\{ \begin{array}{ll} w_{r\_g}*w_{parent}*w_{is\_a}& {t_{i}^{G^{\prime}}=0;}\\ min\left(1,t_{i}^{G^{\prime}}+w_{r\_g}*w_{incre}*w_{is\_a}\right)& {t_{i}^{G^{\prime}}\neq0;} \end{array}\right.  $$
  The more distant from the directly annotated terms, the less relevant the terms are. Therefore, HVSM only considers parent and grandparent terms upward.
iii. Deal with children.
  Only common descendant terms of two or more directly annotated terms are considered, because descendants are less relevant than parents. We use a similar strategy as in step i, replacing parameter *w*_*parent*_ with *w*_*child*_, which corresponds to the semantic contribution factor for child terms. The value of *i*th component corresponding to a child after modification, $t_{i}^{G}$, is expressed as:
  11 $$ {} t_{i}^{G}\,=\, \left\{ \begin{array}{ll} w_{child}*w_{is\_a}& {t_{i}^{G^{\prime}}=0;}\\ min\left(1,t_{i}^{G^{\prime}}+w_{child}*w_{incre}*w_{is\_a}\right)& {t_{i}^{G^{\prime}}\neq0;} \end{array}\right.  $$
iv. Deal with grandchildren.
  A similar strategy as in step iii is used to process the grandchildren. The value of *i*th component corresponding to a grandchild after modification, $t_{i}^{G}$, is expressed as:
  12 $$ t_{i}^{G}= \left\{\begin{array}{ll} w_{r\_g}*w_{child}*w_{is\_a}& {t_{i}^{G^{\prime}}=0;}\\ min\left(1,t_{i}^{G^{\prime}}+w_{r\_g}*w_{child}*w_{incre}*w_{is\_a}\right)& {t_{i}^{G^{\prime}}\neq0;} \end{array}\right.  $$

Stage 3 is similar to stage 2, while the “part_of” relation is considered and $w_{is\_a}$ is replaced by $w_{part\_of}$, where $w_{part\_of}$ corresponds to the semantic contribution factor for the “part_of” relation. There are no “part_of” relations existing in the molecular function ontology.

**Fig. 2:**
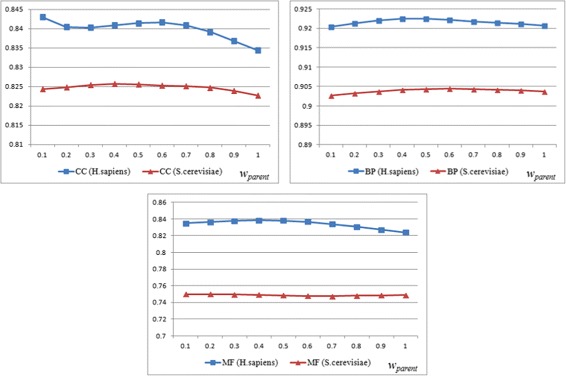
AUC scores for three ontologies over *w*_*parent*_

The semantic contribution of the “part_of” relation is lower than the “is_a” relation [26]. From an intuitive point of view, the parent terms of the directly annotated terms are more relevant than the children terms, parents are more relevant than grandparents, and children are more relevant than grandchildren. This is why $w_{is\_a}>w_{part\_of}$, *w*_*parent*_>*w*_*child*_, and $w_{r\_g}<1$. It is quite complicated to find the optimal combination of all coefficients, for all ontologies and datasets. Especially, the optimal parameters for one ontology may not be optimal for the other ontology. We performed a series of experiments with different coefficient values on the *H. sapiens* and *S. cerevisiae* PPI dataset. One of the experiments was done with different values of *w*_*parent*_. The results are shown in Fig. 2. When *w*_*parent*_=0.5, it was found to have most consistent AUC scores for three ontologies. The set of parameters used in HVSM was the result of trade-offs of all PPI experiments, as shown in Table 1.

**Table 1:** Parameters

$w_{is\_a}$	$w_{part\_of}$	*w*_*parent*_	*w*_*child*_	*w*_*incre*_	$w_{r\_g}$
1	0.7	0.5	0.2	$\frac {1}{6}$	0.5

The similarity measure calculated by VSM is relatively small. Thus, we introduce the concept of a Certainty Factor to calibrate the similarity based on the number of terms annotated to genes. The certainty factor is defined as:

13 $$\begin{array}{@{}rcl@{}} \lambda=\ln(S_{1}+S_{2}) \end{array} $$

where *S*_*i*_ represents the total number of terms annotated to genes. Finally, the similarity between two vectors is defined as:

14 $$\begin{array}{@{}rcl@{}} S_{v}(G_{1},G_{2})=\frac{\lambda(v_{1}\cdot{v_{2}})}{\left|v_{1}\right|\left|v_{2}\right|} \end{array} $$

Because the number of terms associated with genes is very limited, the vectors generated by HVSM are usually quite sparse. When calculating the similarity between two vectors, we remove all the common zero dimensions of two vectors to improve the execution performance of the algorithm.

**Fig. 3:**
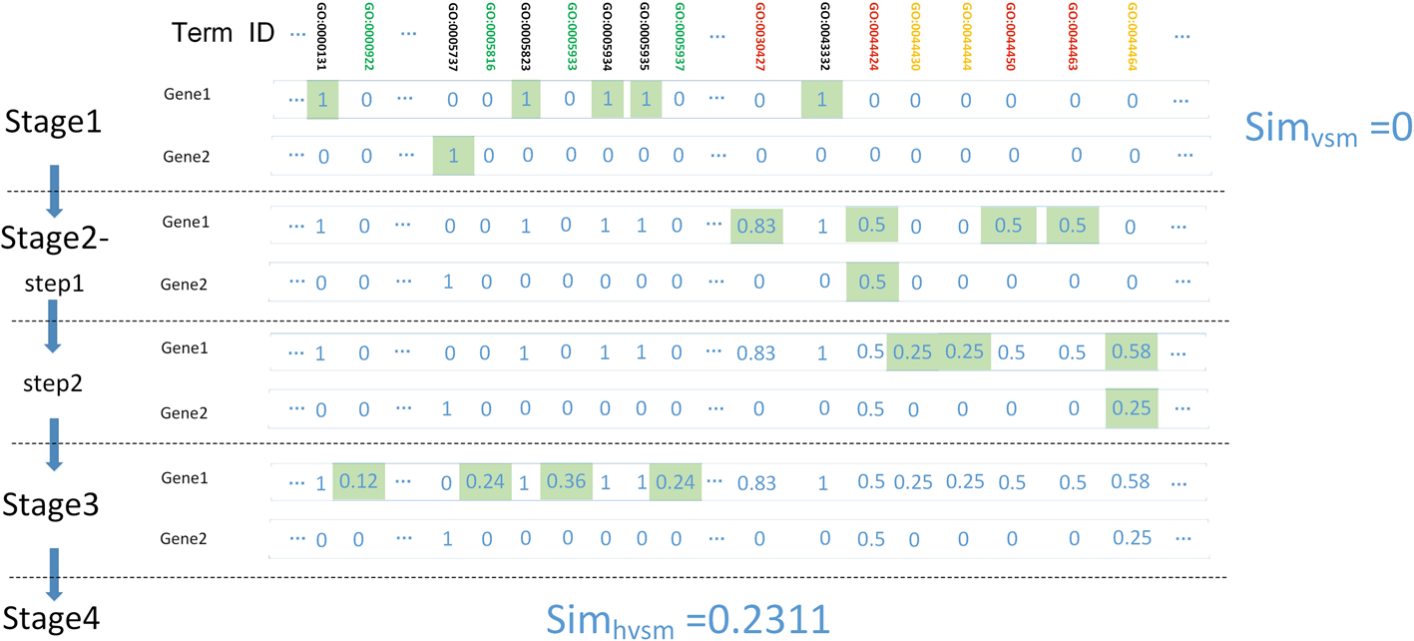
An example illustrating the algorithm. Gene1:S000000313 is annotated to 5 terms and Gene2: S000000825 is annotated to 1 term, which are written in black color. Parent and grandparent terms via “is_a” relations are in red and orange respectively. The terms in green are related via “part_of” relation. Gene1 and Gene2 happen to have no common descendants. The steps in stage3 are not shown because they are almost the same as stage2. The vector components in green background are the ones changed in the steps. The similarity by VSM can be simply calculated from the two vectors in stage1, which is 0. However the gene pair is labeled as positive in the yeast dataset. The similarity obtained by HVSM is 0.23

**Fig. 4:**
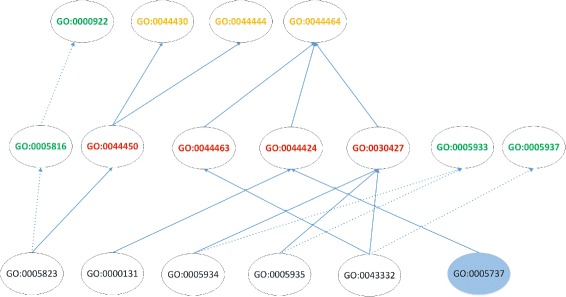
The partial GO topology relevant to the example. Solid lines indicate the “is_a” relation, and dotted lines indicate the “part_of” relation. The term annotated to Gene2 is in blue background

A simple example is provided to illustrate the computation process of HVSM as shown in Fig. 3. The part of GO topology from CC ontology relevant to the example is shown in Fig. 4.

## Results

It is known that comparing the performance of semantic similarity analysis in GO is difficult, because most of the measures use different datasets and different version of ontologies [11, 48]. We used *Homo sapiens* and *Saccharomyces cerevisiae* positive and negative protein interaction sets to evaluate HVSM as a classifier to distinguish positive and negative interactions. We also used Collaborative Evaluation of GO-based Semantic Similarity Measures (CESSM) online tool to compare HVSM to existing measures based on their correlation with sequence, Pfam, and Enzyme Classification similarity.

### Datasets

We adopted the same *Homo sapiens* and *Saccharomyces cerevisiae* PPI datasets and GO annotation file used in Jain, et al. [14]. Ontology data used in our experiments was downloaded from the Gene Ontology database (released in September 2016). The GO contains 29969 BP terms, 4200 CC terms and 11295 MF terms.

Gene annotations for GO terms were downloaded from the Gene Ontology database for H. Sapiens (dated August 2010) [49] and S. cerevisiae (dated February 2010) [50].

The positive and negative protein-protein interaction datasets for H. sapiens and S. cerevisiae were created as follows.

*Homo sapiens*: 2077 unique pairwise PPIs (with three or more publications) for *Homo sapiens* were retrieved from the core set of Database of Interacting Proteins (DIP) (dated June 2010) [51]. The DIP core database records data derived from both small-scale and large-scale experiments that have been validated by the occurrence of the interaction between paralogous proteins in different species [14]. The positive dataset for CC, BP, and MF ontologies comprised interactions with both proteins annotated to terms (other than root) in their respective ontologies. The negative interaction dataset contained an equal number of randomly selected interactions from a pool of all possible interactions in human except for those known to be positive in a set of all known (43,935) human PPIs from iRefWeb [52]. iRefWeb was a meta-database containing the ten largest primary PPI databases [52].

*Saccharomyces cerevisiae*: 4598 unique pairwise *Saccharomyces cerevisiae* PPIs were retrieved from DIP (dated December 2009). The positive dataset for CC, BP, and MF ontologies comprised interactions with both proteins annotated to terms (other than root) in their respective ontologies. The negative dataset with the same number of PPIs as the positive set was generated by randomly selecting proteins from genes in the GO annotation files that are not known to be positive in a set of all known (45,448) yeast PPIs from iRefWeb.

When calculating the similarity on the dataset chosen above at the IntelliGO website (http://plateforme-mbi.loria.fr/intelligo/), we encountered two problems: (1) the corresponding geneid of certain genes from the dataset can not be found in NCBI; (2) a few errors were reported for some gene pairs. To compare the methods fairly, we tested all measures on two sets of data:

1. Use the complete PPI dataset provided in [14]. When the two problems described above occurred, we adopted the processing method used in the HRSS algorithm [53]. When the first problem occurred, the similarity of the gene pair under consideration was set to − 1. When the second problem occurred, the similarity was set to − 2.
2. Use the partial dataset, which means removing the potentially problematic gene data. The negative and positive data distributions of the dataset including or excluding potentially problematic genes are shown in Table 2. The ratio of potentially problematic genes is shown in Tables 3 and 4.

**Table 2:** Negative and positive data distribution before and after the removal

	*S. cerevisiae*				*H. sapiens*			
	Positive		Negative		Positive		Negative	
	Complete dataset	Partial dataset	Complete dataset	Partial dataset	Complete dataset	Partial dataset	Complete dataset	Partial dataset
CC	4469	4248	4469	1859	1431	1422	1431	1176
BP	4385	4123	4385	1750	1435	1424	1435	1177
MF	3858	3641	3858	1589	1441	1407	1441	1167

**Table 3:** Ratio of removed data in the H. sapiens dataset

*H. sapiens*	CC	MF	BP
Positive	0.62%	2.30%	0.70%
Negative	10.80%	19%	17.9%

**Table 4:** Ratio of removed data in the S. cerevisiae dataset

*S. cerevisiae*	CC	MF	BP
Positive	4.90%	5.62%	5.97%
Negative	58.40%	59%	60.1%

Note that more than half of the negative *S. cerevisiae* data have problems. When conducting experiments on the complete dataset, we set the similarity of the gene pairs with problems to either − 1 or − 2. Therefore, the experiment results on the complete *S. cerevisiae* dataset may be unreliable.

### Performance measures

We used the ROC (Receiver Operating Characteristic) curve to evaluate the classification effects of HVSM and other measures for PPI experiments. The ROC curve illustrates the diagnostic ability of a classifier system. The ROC curves are created by plotting *TPR* (true positive rate) against *FPR* (false positive rate). *TPR* and *FPR* are defined as:

15 $$\begin{array}{@{}rcl@{}} TPR=\frac{TP}{(TP+FN)} \end{array} $$

16 $$\begin{array}{@{}rcl@{}} FPR=\frac{FP}{(FP+TN)} \end{array} $$

where *TP*, *TN*, *FP*, and *FN* are the number of True Positive, True Negative, False Positive, and False Negative, respectively. The ideal ROC curve is close to the upper left corner. The closer the ROC curve is to the upper left corner the more accurate the classifier is. Ideally, the area under the ROC curve (AUC) is equal to 1. Therefore, it can be concluded that the larger the *AUC*, the better the classifier is. *AUC* is defined as:

17 $$\begin{array}{@{}rcl@{}} AUC=\frac{1}{2}\sum_{k=1}^{n}\left(X_{k}-X_{k-1}\right)\left(Y_{k}-Y_{k-1}\right) \end{array} $$

where *X*_*k*_ is *FPR*, and *Y*_*k*_ is *TPR*.

To test how our method performs in another application scenario, we tested its correlation using Collaborative Evaluation of GO-based Semantic Similarity Measures (CESSM). CESSM is an online tool [54] that provide a convenient way to compare a specific measure against 11 previously published measures based on their correlation with sequence, Pfam, and Enzyme Classification (EC) similarity. A dataset of 13,430 protein pairs was used involving 1039 unique proteins from various species. Protein pairs (from multiple species), GO (dated August 2010), and UniProt GO annotations (dated August 2008) were downloaded from CESSM. The similarities for the 13,430 proteins pairs were calculated with HVSM and returned to CESSM for evaluation.

### PPI tests

We compared HVSM with the other popular semantic similarity measures, including TCSS [14], IntelliGO [13], basic VSM, Resnik [15], Lin [16], Jiang [17], Schlicker [18], and SimGIC [19], focusing on TCSS. TCSS is widely used and proven to be effective [14] and Resnik is a classic measure. IntelliGO and basic VSM are both vector-based, same as HVSM. The results for *H. sapiens* and *S. cerevisiae* PPI datasets are shown in Tables 5 and 6. The experimental results show that the performance of HVSM is improved up to 12% compared to VSM, 8% compared to IntelliGO, 4% compared to TCSS, 6% compared to Resnik, 8% compared to Lin, 11% compared to Jiang, 8% compared to Schlicker, and 11% compared to SimGIC. Note that the percentage numbers in *the color red* in Table 6 were obtained on the unreliable dataset, as mentioned previously.

**Table 5:** Improvement of HVSM compared with VSM, IntelliGO, TCSS, Resnik, Lin, Jiang, Schlicker, and SimGIC on the H. sapiens PPI datasets

	CC		BP		MF	
	Complete dataset	Partial dataset	Complete dataset	Partial dataset	Complete dataset	Partial dataset
VSM	5%	5%	12%	11%	9%	9%
IntelliGO	3%	3%	2%	2%	6%	8%
TCSS	2%	1%	1%	0%	3%	4%
Resnik	4%	5%	2%	2%	4%	6%
Lin	6%	7%	3%	3%	5%	5%
Jiang	6%	7%	5%	4%	4%	5%
Schlicker	6%	7%	3%	2%	7%	8%
SimGIC	5%	5%	11%	10%	8%	8%

**Table 6:** Improvement of HVSM compared with VSM, IntelliGO, TCSS, Resnik, Lin, Jiang, Schlicker, and SimGIC on the S. cerevisiae PPI datasets

	CC		BP		MF	
	Complete dataset	Partial dataset	Complete dataset	Partial dataset	Complete dataset	Partial dataset
VSM	3%	3%	6%	6%	4%	2%
IntelliGO	*-5%*	2%	*-1%*	7%	*-10%*	2%
TCSS	0%	0%	1%	2%	-1%	-2%
Resnik	0%	0%	2%	2%	0%	-2%
Lin	3%	2%	4%	4%	8%	7%
Jiang	4%	4%	5%	6%	11%	10%
Schlicker	2%	2%	3%	4%	8%	6%
SimGIC	3%	2%	6%	5%	4%	2%

The unreliable results are in italic

#### *Homo sapiens* PPI test

We evaluated the ability of HVSM, TCSS, IntelliGO, VSM and the other five methods to distinguish between the negative and positive using the H. sapiens positive and negative protein interaction sets. Both BMA and MAX approaches were applied to compare with other measures in [14], and MAX was found to have better performance. Therefore, we only compared HVSM with the TCSS MAX approach. TCSS focused on manually annotated GO annotations (“without” annotations with IEA evidence codes (IEA-)), but it was also tested with all annotations, including electronic annotations (“with” annotations with IEA evidence codes (IEA+)). TCSS worked better with (IEA+) than (IEA-). Therefore, we only presented comparison results with (IEA+).

**Fig. 5:**
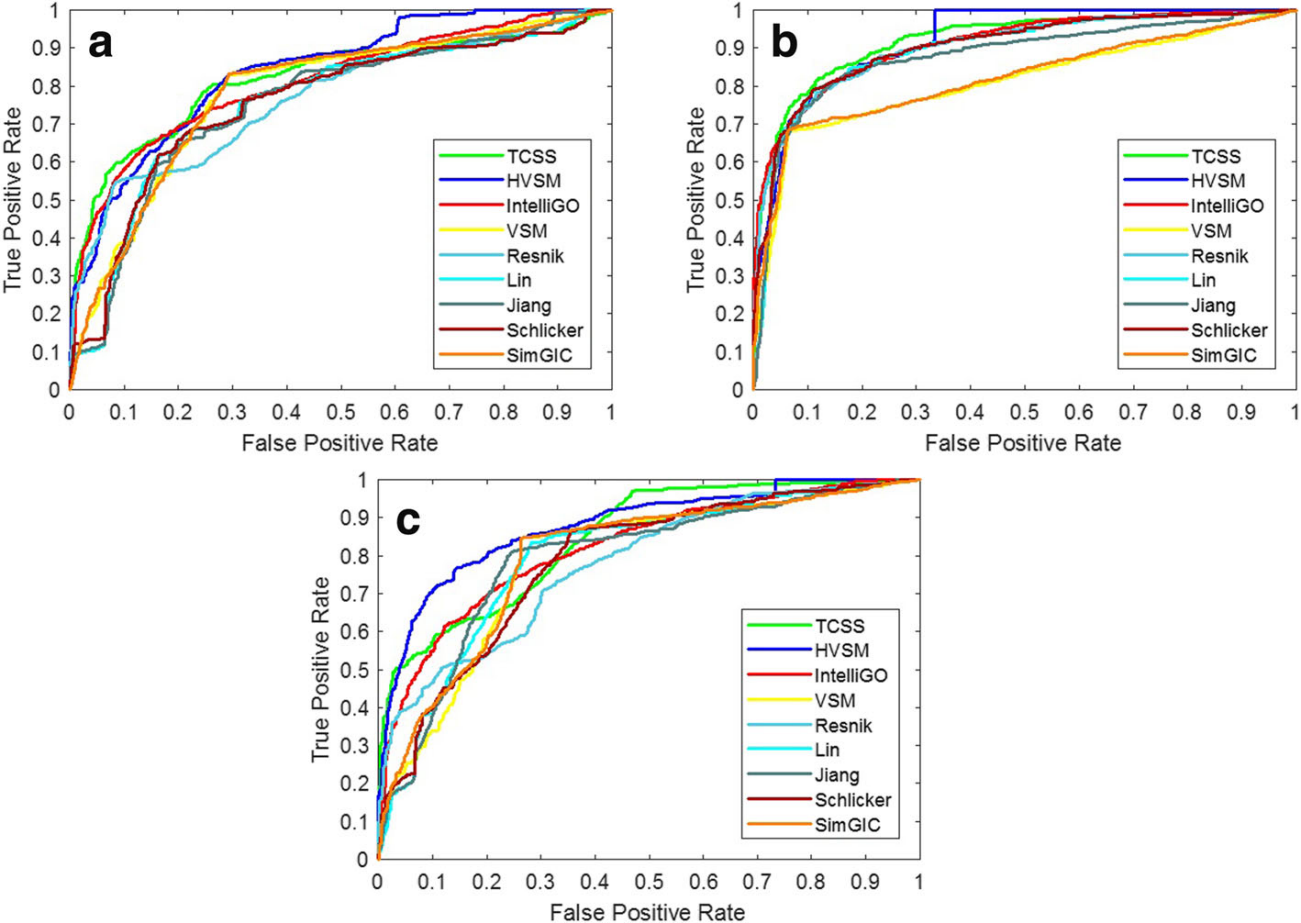
ROC curve on the H. sapiens PPI dataset (Complete dataset). **a** Cellular Component, **b** Biological Process, **c** Molecular Function

**Fig. 6:**
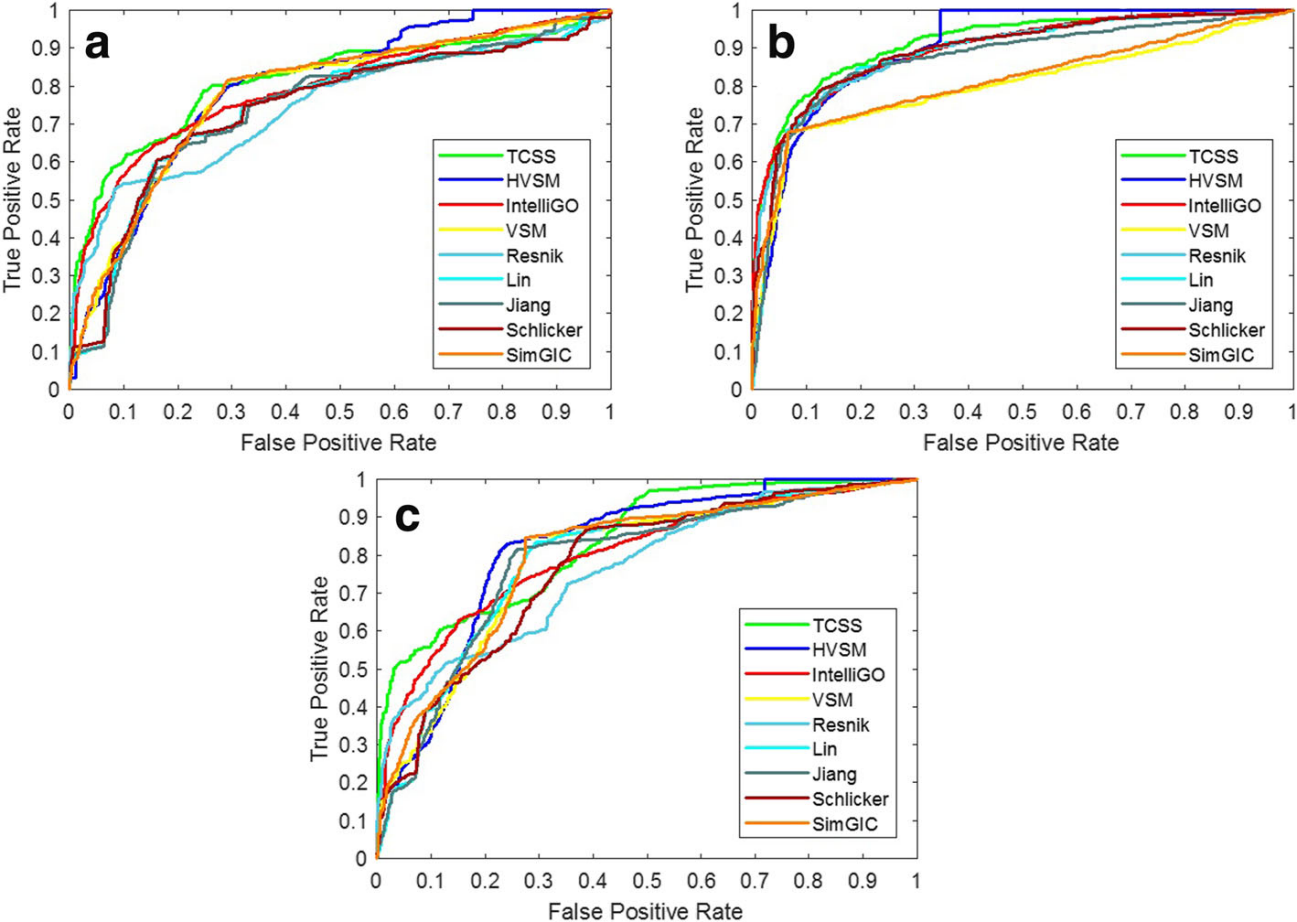
ROC curve on the H. sapiens PPI dataset (Partial dataset). **a** Cellular Component, **b** Biological Process, **c** Molecular Function

Tests were done for CC, BP, and MF ontologies. The AUC scores for the three ontologies are shown in Table 7. HVSM outperforms all other measures in all cases. HVSM performs best for MF ontology on the partial dataset, with an improvement of 4% compared to TCSS, 8% compared to IntelliGO, 9% compared to VSM, 6% compared to Resnik, 5% compared to Lin, 5% compared to Jiang, 8% compared to Schlicker, and 8% compared to SimGIC. No significant performance difference between the complete dataset and partial dataset is observed for the nine measures. The ROC curves are shown in Figs. 5 and 6.

**Table 7:** Area under ROC curves (AUCs) on the H. sapiens PPI dataset

	CC		BP		MF	
	Complete dataset	Partial dataset	Complete dataset	Partial dataset	Complete dataset	Partial dataset
HVSM	**0.84**	**0.83**	**0.93**	**0.92**	**0.88**	**0.88**
TCSS	0.82	0.82	0.92	0.92	0.85	0.84
IntelliGO	0.81	0.80	0.91	0.90	0.82	0.80
VSM	0.79	0.78	0.81	0.81	0.79	0.79
Resnik	0.80	0.78	0.91	0.90	0.84	0.82
Lin	0.78	0.76	0.90	0.89	0.83	0.83
Jiang	0.78	0.76	0.88	0.88	0.84	0.83
Schlicker	0.78	0.76	0.90	0.90	0.81	0.80
SimGIC	0.79	0.78	0.82	0.82	0.80	0.80

The best results are in bold

#### *Saccharomyces cerevisiae* PPI test

**Fig. 7:**
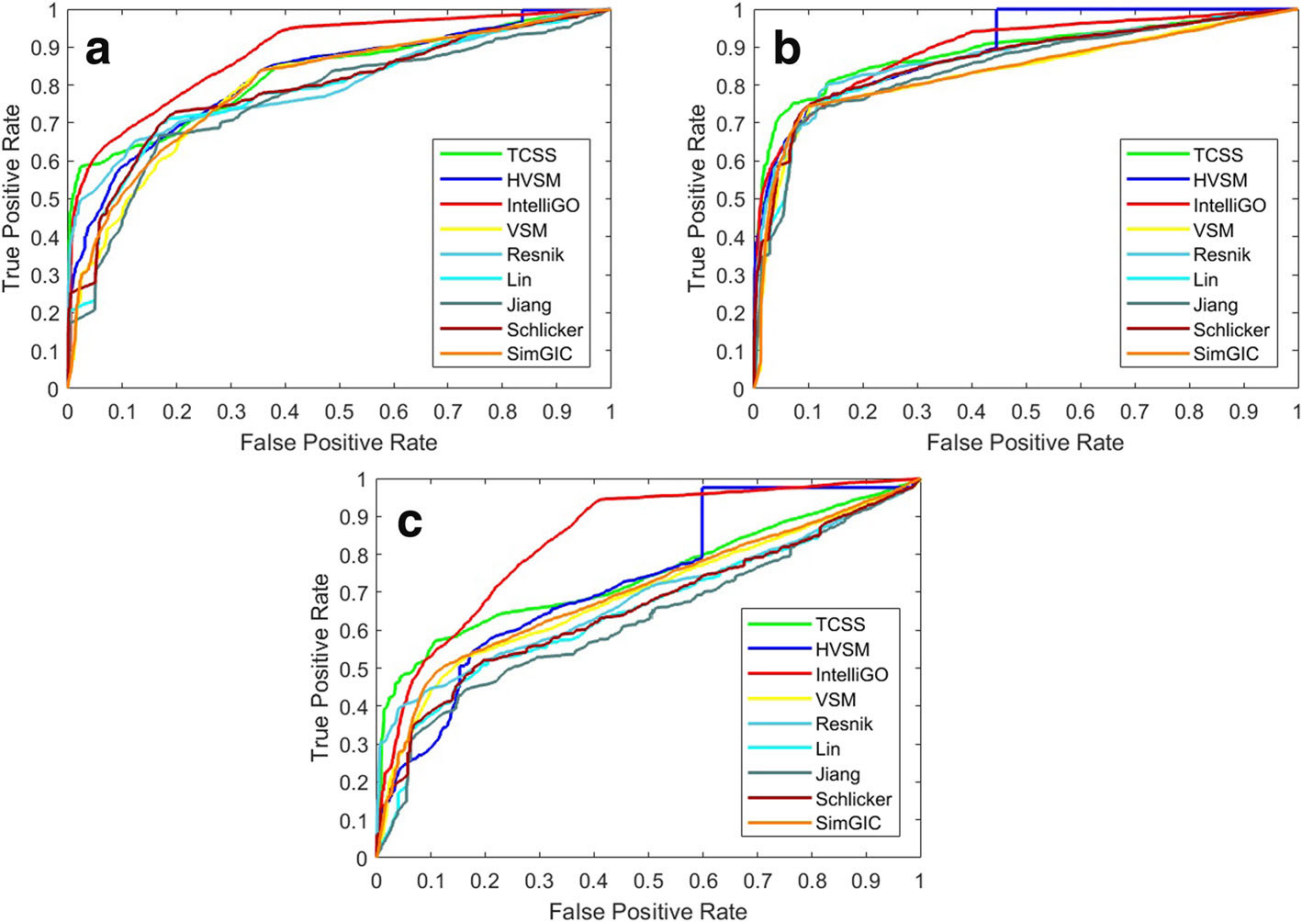
ROC curve on the S.cerevisiae PPI dataset (Complete dataset). **a** Cellular Component, **b** Biological Process, **c** Molecular Function

**Fig. 8:**
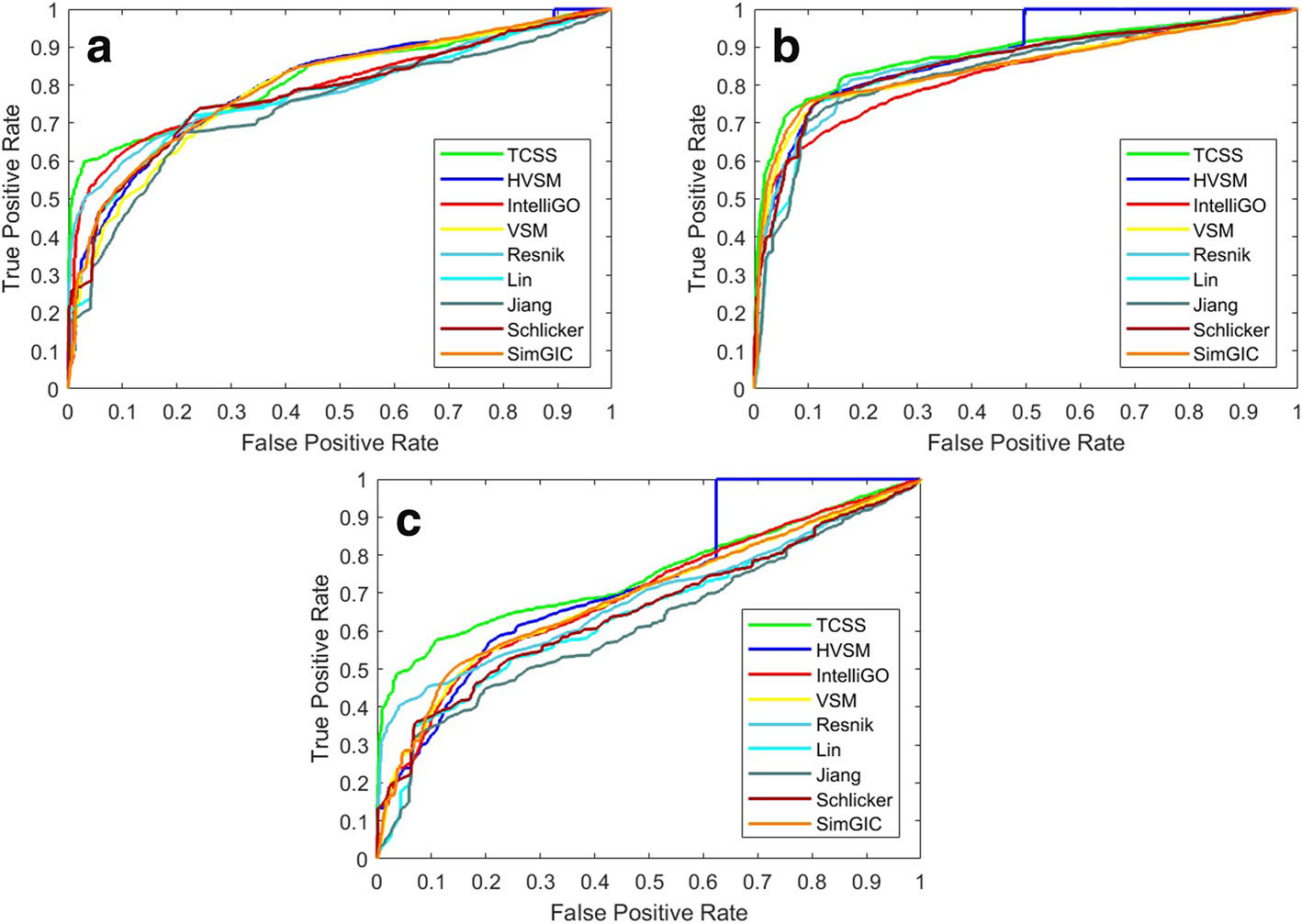
ROC curve on the S.cerevisiae PPI dataset (Partial dataset). **a** Cellular Component, **b** Biological Process, **c** Molecular Function

We applied all nine methods on the *Saccharomyces cerevisiae* PPI datasets. The AUC scores for three ontologies are shown in Table 8. Note that only IntelliGO is sensitive to the problematic dataset, where the performance on the complete dataset is much better than the partial dataset, as shown in Table 8. If we exclude the unreliable IntelliGO results (numbers in *the color red*), HVSM performs best for CC and BP ontology. For MF ontology, HVSM performs only 1% lower than TCSS, similarly to Resnik, and better than VSM and the other five measures. The ROC curves are shown in Figs. 7 and 8.

**Table 8:** Area under ROC curves (AUCs) on the S. cerevisiae PPI dataset

	CC		BP		MF	
	Complete dataset	Partial dataset	Complete dataset	Partial dataset	Complete dataset	Partial dataset
HVSM	**0.83**	**0.82**	**0.90**	**0.90**	0.74	0.72
TCSS	**0.83**	**0.82**	0.89	0.88	**0.75**	**0.74**
IntelliGO	*0.88*	0.80	*0.89*	0.83	*0.84*	0.70
VSM	0.80	0.79	0.84	0.84	0.70	0.70
Resnik	**0.83**	**0.82**	0.88	0.87	0.74	**0.74**
Lin	0.80	0.80	0.86	0.86	0.66	0.65
Jiang	0.79	0.78	0.85	0.84	0.63	0.62
Schlicker	0.81	0.80	0.87	0.86	0.66	0.66
SimGIC	0.80	0.80	0.84	0.85	0.70	0.70

The unreliable results are in italic. The best results are in bold

### CESSM test

HVSM measure was used to calculate similarities for the benchmark set of protein pairs downloaded from the CESSM website [54]. The benchmark set represents three different types of similarities, based on sequence similarity, Enzyme Classification (EC), and protein domains (Pfam). We compared HVSM with our main concern TCSS and four other measures provided by CESSM: Resnik, Lin, Jiang, and SimGIC. MAX approach was selected for Resnik, Lin, and Jiang. The results obtained (correlation coefficients) are presented in Table 9. HVSM is superior to all measures except for SimGIC. The HVSM correlation coefficient for the EC dataset is higher than all other methods. For the Pfam dataset, HVSM is comparable to SimGIC. For the sequence dataset, the value obtained with HVSM is beaten by SimGIC, but better than all other measures. One cause for this could be that SimGIC scores gene products with shared annotation terms. Since gene products annotated to same term are more likely to be part of the same gene family and thus SimGIC has high sequence similarity [14]. HVSM performs better for CC and BP ontology than MF ontology. For CESSM, we fine-tuned the parameters based on the values in Table 1 by adjusting *w*_*child*_ to 0.05.

**Table 9:** Results obtained with CESSM

		Methods					
		HVSM	TCSS	Resnik	Lin	Jiang	SimGIC
CC	EC	**0.36**	0.33	0.29	0.26	0.18	0.36
	Pfam	0.47	0.45	0.38	0.35	0.21	**0.50**
	Sequence	0.67	0.62	0.48	0.42	0.33	**0.75**
BP	EC	**0.43**	0.16	0.31	0.31	0.25	0.40
	Pfam	**0.47**	0.12	0.26	0.21	0.17	0.46
	Sequence	0.75	0.28	0.30	0.25	0.24	**0.77**
MF	EC	**0.71**	0.61	0.45	0.45	0.36	0.62
	Pfam	0.46	0.41	0.18	0.18	0.13	**0.64**
	Sequence	0.38	0.48	0.13	0.12	0.10	**0.72**

The best results are in bold

## Discussion

Our experiments showed that the results with the confidence factor were significantly better than those without it. It can be proved that the relative value of similarities of pairs of genes are not affected by the base of the logarithm in equation (13), as long as the base is greater than 1. In other words, the base of the logarithm does not change the order of the similarity ranking. Hence, the base of the logarithm of the confidence factor does not affect the ROC analysis results. Since the multiplication of the confidence factor may cause the similarity values calculated by HVSM to be greater than 1, a single similarity value could not be used directly. This problem does not affect the effectiveness of HVSM as a classifier to distinguish positive and negative interactions. In any case, a proper normalization method needs to be investigated in the future.

The coefficients used in HVSM, such as $w_{is\_a} w_{part\_of} w_{parent}$ and *w*_*child*_, were decided by the intuitive speculation and the experiments on the *H. sapiens* and *S. cerevisiae* PPI datasets. We have tried to look for the optimal combination of the five coefficients for all datasets and ontologies. Right now they are the results of approximate trade-offs and may not be the best answer. More experiments and datasets should be tested. The alternative way is to find different combinations for different ontologies or datasets.

## Conclusions

We presented a new method to calculate semantic similarity, the Hierarchical Vector Space Model, which enhanced the basic vector space model by introducing the relations between GO terms. When constructing the gene vector, we took into account the terms related by two types of relations: “is_a” and “part_of”. Moreover, HVSM introduced the concept of the Certainty Factor to calibrate the similarity based on the number of annotated terms.

To assess the effectiveness of HVSM, we performed experiments using *H. sapiens* and *S. cerevisiae* protein-protein interaction datasets, and compared the results with TCSS, IntelliGO, basic VSM, Resnik, Lin, Jiang, Schlicker, and SimGIC measures. The results showed that HVSM outperformed the other eight measures in most cases. HVSM achieved an improvement of up to 4% compared to TCSS, 8% compared to IntelliGO and 12% compared to VSM, 6% compared to Resnik, 8% compared to Lin, 11% compared to Jiang, 8% compared to Schlicker, and 11% compared to SimGIC. We also tested the correlation between multiple semantic similarity scoring methods with sequence, EC, and Pfam similarity with CESSM. The results showed that HVSM was a comparable measure relative to SimGIC, and outperformed all other similarity measures in CESSM as well as TCSS.

## Abbreviations

BP: Biological Process
CC: Cellular Component
CESSM: Collaborative Evaluation of GO-based Semantic Similarity Measures
DAG: Directed Acyclic Graph
EC: Enzyme Classification
FN: False Negative
FP: False Positive
FPR: False Positive Rate
GO: Gene Ontology
HVSM: Hierarchical Vector Space Model
IC: Information Content
LCA: Lowest Common Ancestor
MF: Molecular Function
ROC: Receiver Operating Characteristic
TN: True Negative
TP: True Positive
TPR: True Positive Rate
VSM: Vector Space Model
